# Accelerated Plethysmography in Glaucoma Patients

**DOI:** 10.3390/biomedicines13071542

**Published:** 2025-06-24

**Authors:** Hinako Takei, Yuto Yoshida, Misaki Ukisu, Keigo Takagi, Masaki Tanito

**Affiliations:** Department of Ophthalmology, Shimane University Faculty of Medicine, Izumo 693-8501, Japan

**Keywords:** accelerated plethysmography, primary open angle glaucoma, exfoliation glaucoma, vascular stiffness, atherosclerosis

## Abstract

**Background:** Systemic arterial stiffness and atherosclerosis have been increasingly recognized as potential contributors to the pathogenesis of glaucoma. Several studies have reported associations between glaucoma and various surrogate markers of vascular stiffness. However, despite the growing interest in the vascular components of glaucoma, no previous studies have specifically explored the relationship between the indices derived from acceleration plethysmography (APG) and glaucoma. This study seeks to address this gap by investigating the potential association between APG parameters and the presence of glaucoma. **Methods:** The subjects were 701 patients (mean age 68.6 years, 54% male) with open-angle glaucoma (primary open-angle glaucoma [POAG] or exfoliation glaucoma [EXG]), and 94 control subjects (mean age 60.1 years, 57% male) who had no eye diseases other than cataracts. The subjects were all cases in which APG was measured using a sphygmograph (TAS9 Pulse Analyzer Plus View; YKC Corp., Tokyo, Japan). The amplitude of waveform types (a, b, c, d, and e-waves) and derived vascular types (A, B, and C) of the accelerated pulse wave components were statistically compared between the cases and controls. **Results:** The accelerated pulse wave components (mean ± standard deviation) of the control and glaucoma groups were a-wave 785 ± 99 and 776 ± 93 (*p* = 0.40), b-wave −522 ± 161 and −491 ± 143 (*p* = 0.050), c-wave −142 ± 108 and −156 ± 105 (*p* = 0.24), d-wave −288 ± 144 and −322 ± 122 (*p* = 0.014), and e-wave 103 ± 79 and 90 ± 58 (*p* = 0.059), with differences between the groups being observed in the b and d-waves. For derived vascular types, compared with the controls and POAG, patients with EXG had a lower frequency of Type A and a higher frequency of Type C than the other groups (*p* = 0.044). Multivariate analysis showed that factors significantly associated with vascular type included age (*p* < 0.0001), sex (*p* < 0.0001), diastolic blood pressure (*p* = 0.021), and pulse rate (*p* < 0.0001), while BMI, systolic blood pressure, history of hypertension, history of diabetes, presence or absence of glaucoma, and presence or absence of pseudoexfoliation material were not significant. **Conclusions:** This is the first study to investigate the relationship between APG and glaucoma with a large sample size. In elderly glaucoma patients, particularly those with EXG, systemic vascular changes are often present. APG parameters may reflect vascular alterations in glaucoma.

## 1. Introduction

Glaucoma is a leading cause of irreversible blindness worldwide [1]. With the global population aging rapidly, the number of individuals affected by glaucoma is projected to rise to 111.8 million by 2040 [2]. Lowering intraocular pressure (IOP) remains the most established and evidence-based strategy for preventing disease progression [3]. Additionally, several other factors have been implicated in the development of glaucoma, including myopia [4], oxidative stress [5], ocular blood flow [6], and autonomic nervous system dysfunction [7,8,9].

Glaucoma may also be associated with systemic arterial stiffness and atherosclerosis. A previous study demonstrated that patients with glaucoma exhibited reduced brachial flow-mediated dilation (FMD), indicating the presence of systemic endothelial dysfunction [10]. Lee et al. reported that elevated pulse wave velocity (PWV) had been associated with reduced macular vessel density in patients with normal-tension glaucoma (NTG), suggesting that systemic arterial stiffness may contribute to the pathogenesis of NTG [11]. Furthermore, several studies reported associations between glaucoma and other markers of arterial stiffness, including the cardio–ankle vascular index (CAVI) [12] and augmentation index (AI) [13].

Among these vascular aging indices, acceleration plethysmography (APG)—the second derivative of the photoplethysmogram—has been widely used to evaluate arterial stiffness and vascular aging [14]. APG-derived parameters, such as the relative amplitudes of waveform components (a, b, c, d, and e) or composite vascular aging indices, are established noninvasive markers in cardiovascular research [15]. However, no studies have examined the association between APG-derived indices and glaucoma. Given these knowledge gaps, this study aims to investigate the potential association between APG and glaucoma.

## 2. Subjects and Methods

### 2.1. Study Design and Subjects

We conducted a retrospective cross-sectional study based on medical record information. This study followed the principles outlined in the Declaration of Helsinki and the Ethical Guidelines for Medical and Health Research Involving Human Subjects in Japan. The Institutional Review Board (IRB) of Shimane University Hospital reviewed and approved the research (No. 20200228-2, revised version issued on 27 October 2024). The IRB approval did not necessitate written informed consent from each patient for publication. Instead, the study protocol was made available at the study institutions, allowing participants to opt-out if desired. Subjects were recruited consecutively at the Department of Ophthalmology, Shimane University Hospital, spanning from June 2023 to July 2024. The study included patients with open-angle glaucoma [primary open-angle glaucoma (POAG) or exfoliation glaucoma (EXG)], as well as individuals without ocular diseases other than cataracts (control group). The inclusion criteria encompassed all individuals who underwent vascular assessment using a pulse analyzer. The exclusion criteria were: (1) a measurement reliability score of less than 95% and (2) the presence of ocular diseases other than OAG or cataracts.

### 2.2. Accelerated Plethysmography (APG)

APG was obtained from the participants’ fingertips using a sphygmograph (TAS9 Pulse Analyzer Plus View; YKC Corp., Tokyo, Japan) set to APG measurement mode. The recordings were acquired at a sampling rate of 1 kHz. During the measurement, a sensor was affixed to the index finger of the left hand, with the finger maintained at approximately the same height as the heart to ensure accurate readings. Then, the changes in fingertip (peripheral) blood vessel volume due to heartbeat, known as photoplethysmography (PPG), were depicted as a waveform. APG was derived by calculating the second derivative of the PPG waveform. All APG measurements were performed by experienced ophthalmic technicians.

**Figure 1:**
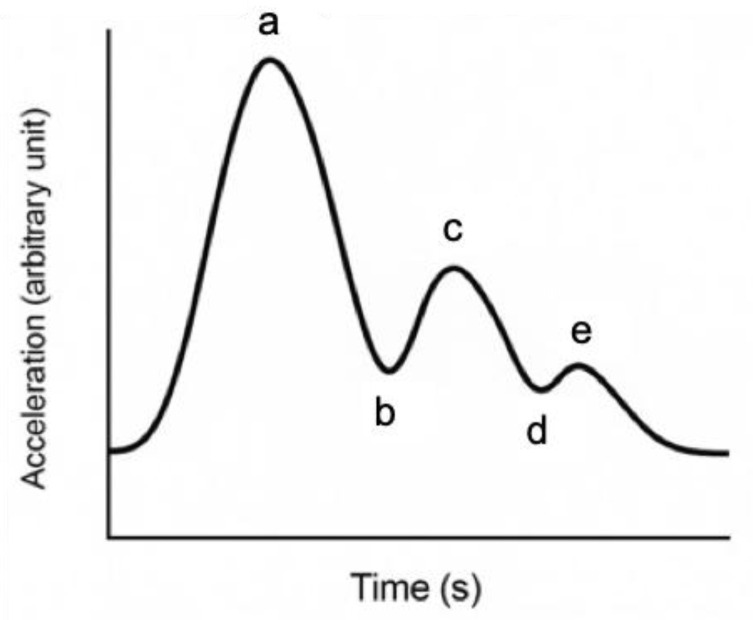
Schematic diagram of APG waveform (a–e). For descriptions of each wave, see main text. Images generated by Chat GPT-4o.

**Figure 2:**
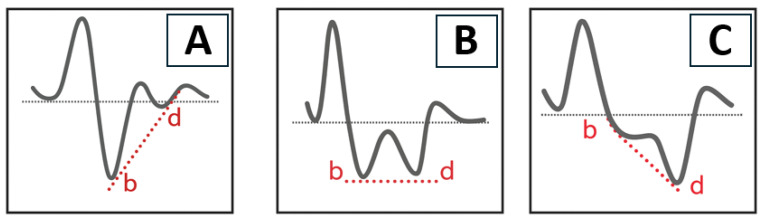
Schematic diagram of vascular types (**A**–**C**). The dotted line represents a line connecting the peaks of the b-wave and d-wave. For descriptions of each type, see main text. Images generated by Chat GPT-4o.

The APG waveform consists of five components, labeled a through e (Figure 1), each reflecting different phases of arterial pulse dynamics. Among four normal waveform data points, the highest and lowest values are excluded, and the average of the remaining two values is analyzed at designated points. The a-wave corresponds to the initial systolic upstroke caused by left ventricular ejection. The b-wave reflects the early systolic reflection wave from peripheral arteries, typically prominent in young, healthy arteries. The c-wave represents the late systolic component, while the d-wave is associated with diastolic reflection and tends to increase with arterial stiffness. The e-wave indicates the end-diastolic phase and may vary depending on peripheral vascular resistance [16]. The APG waveform was categorized into three vascular types (A, B, and C) based on the amplitude and morphology of the a–e waves (Figure 2). Type A represents elastic and healthy arteries, typically showing a well-defined b-wave. Type B reflects moderate arterial stiffness, with a reduced b-wave and an increased d-wave. Type C indicates advanced arterial stiffness, characterized by the disappearance of the b-wave and dominance of the d-wave.

### 2.3. Other Covariates

In this study, we collected the following variables: age, sex, body mass index (BMI), systolic blood pressure (sBP), diastolic blood pressure (dBP), pulse rate, and presence of hypertension and diabetes mellitus (DM). Ophthalmic parameters extracted from the records included the presence or absence of glaucoma and pseudoexfoliation material (PEM). In this study, eyes in which both glaucoma and PEM were identified were classified as having exfoliation glaucoma (EXG).

### 2.4. Statistical Analysis

The data were statistically analyzed separately for those who were with glaucoma and those who were not. The data were presented as mean ± standard deviation (SD) with 95% confidence interval (CI) ranges for continuous parameters and as numbers and percentages for categorical parameters. The potential association between patients with glaucoma and those without glaucoma was evaluated using an unpaired *t*-test for continuous parameters and Fisher’s exact probability test for categorical parameters. The difference in APG wave amplitude between patients with glaucoma and those without glaucoma was evaluated using an unpaired *t*-test. Additionally, multivariate analysis for possible parameters associated with each peak amplitude was conducted using a generalized regression model. The relationship between vascular types and glaucoma was analyzed using an ordinal logistic regression model. Confounders included in the models were age, sex, BMI, sBP, dBP, pulse rate, hypertension, DM, PEM deposition, and presence or absence of glaucoma. All statistical analyses were conducted using the JMP Pro statistical software version 17.2.0 (SAS Institute, Inc., Cary, NC, USA). A *p*-value of less than 0.05 was considered statistically significant.

## 3. Results

A comparison of the demographic characteristics between the glaucoma and non-glaucoma groups is presented in Table 1. The glaucoma group consisted of 701 subjects, and the non-glaucoma group consisted of 94 subjects. There was a significant difference in age between the glaucoma group (mean age ± SD: 68.6 ± 12.5 years) and the non-glaucoma group (60.1 ± 18.6 years). Additionally, significant differences were observed in the pulse rate and the presence of hypertension between the two groups. There were no significant differences in BMI, sBP, dBP, or the presence of DM. Among the 701 subjects in the glaucoma group, 186 (26.5%) had a PEM deposition (i.e., EXG).

Table 2 summarizes the comparisons in the amplitudes of the different APG waveforms between the glaucoma and non-glaucoma groups. Among the APG waves, the b and d peaks showed significant differences between the two groups. The b peak amplitude was −491 ± 143 in the glaucoma group and −522 ± 161 in the non-glaucoma group (*p* = 0.050), while the d peak amplitude was −322 ± 122 and −288 ± 144, respectively (*p* = 0.014). No significant differences were observed in the a, c, and e peak amplitudes between the two groups.

Table 3 shows the multivariate analysis for possible parameters associated with a peak amplitude. Female sex (*p* = 0.031), higher sBP (*p* = 0.015), and the absence of PEM deposition (*p* = 0.047) were associated with a lower peak amplitude. Lower BMI (*p* < 0.0001) and lower pulse rate (*p* = 0.0012) were associated with a decreased peak amplitude. Whether subjects had glaucoma not showed no significant differences.

Table 4 shows the multivariate analysis for possible parameters associated with b peak amplitude. Lower sBP (*p* = 0.0426) and the absence of hypertension (*p* = 0.0472) were associated with a lower peak amplitude. Lower age (*p* < 0.0001), male sex (*p* < 0.0001), higher BMI (*p* < 0.0001), and lower pulse rate (*p* < 0.0001) were strongly associated with a decreased peak amplitude. Whether subjects had a PEM deposition or glaucoma showed no significant differences.

Table 5 shows the multivariate analysis for possible parameters associated with c peak amplitude. Female sex (*p* = 0.020) was associated with a lower peak amplitude. Older age (*p* < 0.0001), BMI (*p* = 0.0028), higher dBP (*p* = 0.0006), lower sBP (*p* < 0.0001), and higher pulse rate (*p* < 0.0001) were strongly associated with a decreased peak amplitude. Whether subjects had a PEM deposition or glaucoma showed no significant differences.

Table 6 shows the multivariate analysis for possible parameters associated with d peak amplitude. Female sex (*p* = 0.033), hypertension (*p* = 0.022), and PEM deposition (*p* = 0.049) were associated with a lower peak amplitude. Older age (*p* < 0.0001), lower sBP (*p* = 0.0091), higher dBP (*p* < 0.0001), and lower pulse rate (*p* = 0.0009) were strongly associated with a decreased peak amplitude. Whether subjects had glaucoma or not showed no significant differences.

Table 7 shows the multivariate analysis for possible parameters associated with e peak amplitude. Lower BMI (*p* = 0.021) and lower dBP (*p* = 0.049) were associated with a lower peak amplitude. Older age (*p* < 0.0001), higher sBP (*p* = 0.0019), lower pulse rate (*p* < 0.0001), and hypertension (*p* = 0.0022) were strongly associated with a decreased peak amplitude. Whether subjects had PEM deposition or glaucoma showed no significant differences.

There was no significant difference in vascular type between the Glaucoma (+) and Glaucoma (−) groups (*p* = 0.32). However, when the Glaucoma (+) group was subdivided into PEM (+) (i.e., POAG) and PEM (−) (i.e., EXG), the EXG group showed a lower frequency of Type A, and a higher frequency of Type C compared to the other groups (*p* = 0.044) (Table 8). The factors associated with vascular type were analyzed using ordinal logistic regression. Older age (*p* < 0.0001), female sex (*p* < 0.0001), higher diastolic blood pressure (*p* = 0.021), and lower pulse rate (*p* < 0.0001) were significantly associated with more arteriosclerotic vascular types (Table 9).

## 4. Discussion

This study is the first study to investigate the association between APG and glaucoma. Among APG waveform components, the b and d peaks showed significant differences between the glaucoma and non-glaucoma groups. In the analysis of vascular waveform types, the EXG group demonstrated a significantly lower frequency of Type A, and a higher frequency of Type C compared to other groups. However, multivariate analyses revealed that neither the APG waveform components nor the vascular types were independently associated with glaucoma.

In the present study, significant differences were observed in the amplitudes of the b and d-waves in the APG between the glaucoma and non-glaucoma groups. As shown in Table 2, the b-wave was shallower in the glaucoma group (−491 ± 143) than in the non-glaucoma group (−522 ± 161; *p* = 0.050), while the d-wave was deeper in the glaucoma group (−322 ± 122) than in the non-glaucoma group (−288 ± 144; *p* = 0.014). These findings may suggest increased arterial stiffness in patients with glaucoma and are consistent with previous studies [16,17,18,19]. Several studies have reported a significant association between open-angle glaucoma and systemic arteriosclerosis [16,17,18]. Moreover, a previous cohort study suggested that increased arterial stiffness may contribute to the development of glaucoma [19]. Given its noninvasive nature, APG may serve as a potential vascular biomarker for glaucoma; however, further studies are needed to validate its utility.

Patients with EXG may exhibit greater arterial stiffness compared to those with POAG or individuals without glaucoma. As shown in Table 8, the EXG group demonstrated a significantly lower incidence of Type A, and a higher incidence of Type C in comparison with the other subgroups (*p* = 0.044). According to the multivariate analysis presented in Table 3 and Table 6, a and d peak amplitudes were independently associated with PEM deposition (*p* = 0.047 and 0.049, respectively). A previous study has demonstrated that patients with EXG exhibit increased arterial stiffness and reduced carotid artery distensibility, suggesting potential systemic vascular involvement in this glaucoma subtype [20]. A cross-sectional study showed that patients with EXG had significantly higher carotid–femoral pulse wave velocity (CF–PWV) values, indicating increased arterial stiffness [21]. However, prior studies on arterial stiffness in those with EXG were limited by small sample sizes. In contrast, the present study was able to evaluate vascular characteristics in a larger EXG population, strengthening the evidence for a link between EXG and systemic arteriosclerosis. Future longitudinal studies are needed to clarify the causal relationship between arterial stiffness and EXG. In the present study, the differences between the glaucoma and non-glaucoma groups became less evident after adjusting for age. This may reflect the higher prevalence of glaucoma, particularly EXG, among older individuals. Given the age-related nature of glaucoma, vascular aging, including arterial stiffness, may also be more common in this population. Further studies are warranted to elucidate the association between glaucoma and systemic vascular aging.

In this study, Table 3, Table 4, Table 5, Table 6 and Table 7 present the multivariate analyses of possible parameters associated with a–e peak amplitudes. These analyses identified significant associations with age, sex, BMI, BP, pulse rate, and hypertension. Additionally, in the multivariate analysis for possible parameters associated with vascular types, age, sex, dBP, and pulse rate were found to be significantly associated. These findings are consistent with previous research. A previous study demonstrated that in women, lower heart rate was significantly associated with higher augmentation pressure and augmentation index, whereas in men, higher heart rate was significantly associated with increased carotid–femoral PWV [22]. The association between APG parameters and these variables, such as age, blood pressure, and heart rate, warrants further investigation.

This study has several limitations. First, the cross-sectional design limits causal interpretation between APG parameters and glaucoma. Second, the sample included significantly older subjects in the glaucoma group, which could have biased APG results due to age-related vascular stiffening. Although statistical adjustments were made, residual confounding may persist. Third, we did not assess ocular blood flow directly, limiting the ability to connect APG findings with intraocular hemodynamics. Fourth, the absence of several factors known to influence vascular stiffness—such as ischemic heart disease, cerebrovascular disease, smoking status, serum lipid levels, and systemic medications—from the analysis represents another limitation of the present study. Fifth, most of the APG measurements were conducted during morning outpatient clinic hours; however, it is important to note that the measurement times were not strictly standardized. In the present study, to ensure measurement accuracy, we excluded any results with a reliability index below 95%. However, we did not evaluate the reproducibility of the measurements. We believe that the inclusion of a large number of cases has helped to normalize the impact of measurement variability. Lastly, the study relied on retrospective data from a single center, which may limit generalizability. Our results clearly demonstrate that systemic arteriosclerotic changes are more advanced in elderly glaucoma patients and those with EXG. These findings suggest the potential importance of focusing not only on ocular factors but also on vascular characteristics in the future prevention and management of glaucoma.

## 5. Conclusions

To our knowledge, this is the first study to explore the relationship between APG and glaucoma with a large sample size. Significant differences in b and d-wave amplitudes were observed between patients with glaucoma and the control subjects. The distribution of vascular waveform types differed significantly between patients with EXG and those with other glaucoma subtypes or controls. These findings suggest that APG may reflect vascular alterations in glaucoma.

## Figures and Tables

**Table 1:** Comparison of demographic data between Glaucoma (+) and Glaucoma (−) groups.

Parameters	Glaucoma (+)		Glaucoma (−)		*p*-Value
	N or Mean ± SD	% or 95% CI	N or Mean ± SD	% or 95% CI	
Subjects	701		94		
Eyes	701		94		
Age, years	68.6 ± 12.5	67.6, 69.5	60.1 ± 18.6	56.3, 63.9	<0.0001 **
Sex					
Male	381	54.3	54	57.4	0.58
Female	320	45.7	40	42.6	
BMI, kg/m^2^	22.7 ± 3.3	22.5, 23.0	23.2 ± 4.4	22.3, 24.1	0.24
sBP, mmHg	142.7 ± 20.8	141.1, 144.3	141.3 ± 23.5	136.3, 146.3	0.57
dBP, mmHg	80.5 ± 13.3	79.5, 81.6	79.5 ± 14.4	76.4, 82.5	0.47
Pulse rate, bpm	73.6 ± 12.5	72.6, 74.5	80.3 ± 15.7	77.0, 83.6	<0.0001 **
Hypertension					
yes	314	44.8	28	29.8	0.006 **
no	387	55.2	66	70.2	
DM					
yes	96	13.7	20	21.3	0.06
no	605	86.3	74	78.7	
PEM deposition					
yes	186	26.5	0	0	<0.0001 **
no	515	73.5	94	100	

*p*-values were calculated using the unpaired *t*-test or Fisher’s exact test. ** *p* < 0.01. BMI, body mass index; sBP, systolic blood pressure; dBP, diastolic blood pressure; bpm, beats per minute; DM, diabetes mellitus; PEM, pseudoexfoliation material; SD, standard deviation; CI, confidence interval.

**Table 2:** Comparison of APG wave amplitudes between Glaucoma (+) and Glaucoma (−) groups.

Parameters	Glaucoma (+)		Glaucoma (−)		*p*-Value
	Mean ± SD	95% CI	Mean ± SD	95% CI	
a peak	776 ± 93	769, 783	785 ± 99	765, 805	0.40
b peak	−491 ± 143	−501, −480	−522 ± 161	−555, −489	0.050 *
c peak	−155 ± 105	−163, −148	−142 ± 108	−164, −120	0.24
d peak	−322 ± 122	−331, −313	−288 ± 144	−318, −259	0.014 *
e peak	90 ± 58	86, 94	103 ± 80	87, 119	0.06

*p*-values were calculated using the unpaired *t*-test. * *p* < 0.05. APG, accelerated plethysmography; SD, standard deviation; CI, confidence interval.

**Table 3:** Multivariate analysis for possible parameters associated with a peak amplitude.

Parameters	Estimate	95% CI	*p*-Value
Age, years	−0.35	0.09, 0.18	0.20
Sex, F/M	−15	−28, −1.4	0.031 *
BMI, kg/m^2^	4.3	2.3, 6.2	<0.0001 **
sBP, mmHg	−0.61	−1.1, −0.12	0.015 *
dBP, mmHg	0.24	−0.51, 0.99	0.53
Pulse rate, bpm	0.86	0.34, 1.4	0.0012 **
Hypertension, yes/no	2.8	−11, 17	0.69
DM, yes/no	1.3	−18, 20	0.90
PEM deposition, yes/no	17.0	0.2, 33	0.047 *
Glaucoma, yes/no	0.32	−22, 23	0.98

*p*-values were calculated using the generalized regression model. ** *p* < 0.01, * *p* < 0.05. BMI, body mass index; sBP, systolic blood pressure; dBP, diastolic blood pressure; bpm, beats per minute; DM, diabetes mellitus; PEM, pseudoexfoliation material; CI, confidence interval.

**Table 4:** Multivariate analysis for possible parameters associated with b peak amplitude.

Parameters	Estimate	95% CI	*p*-Value
Age, years	1.9	1.1, 2.7	<0.0001 **
Sex, F/M	65	44, 86	<0.0001 **
BMI, kg/m^2^	−7.0	−10, −4.0	<0.0001 **
sBP, mmHg	0.70	0.023, 1.4	0.043 *
dBP, mmHg	0.75	−0.40, 1.9	0.20
Pulse rate, bpm	−2.6	−3.4, −1.8	<0.0001 **
Hypertension, yes/no	22	0.27, 43	0.047 *
DM, yes/no	−5.2	−32, 22	0.70
PEM deposition, yes/no	12	−16, 39	0.40
Glaucoma, yes/no	−11	−43, 22	0.51

*p*-values were calculated using the generalized regression model. ** *p* < 0.01, * *p* < 0.05. BMI, body mass index; sBP, systolic blood pressure; dBP, diastolic blood pressure; bpm, beats per minute; DM, diabetes mellitus; PEM, pseudoexfoliation material; CI, confidence interval.

**Table 5:** Multivariate analysis for possible parameters associated with c peak amplitude.

Parameters	Estimate	95% CI	*p*-Value
Age, years	−2.7	−3.3, −2.1	<0.0001 **
Sex, F/M	−17	−32, −2.7	0.020 *
BMI, kg/m^2^	−3.7	−6.1, −1.3	0.0028 **
sBP, mmHg	1.4	0.91, 1.8	<0.0001 **
dBP, mmHg	−1.2	−1.9, −0.51	0.0006 **
Pulse rate, bpm	−1.6	−2.2, −1.1	<0.0001 **
Hypertension, yes/no	−13	−28, 2.8	0.11
DM, yes/no	−5.5	−25, 14	0.58
PEM deposition, yes/no	−7.2	−26, 11	0.45
Glaucoma, yes/no	10	−12, 32	0.38

*p*-values were calculated using the generalized regression model. ** *p* < 0.01, * *p* < 0.05. BMI, body mass index; sBP, systolic blood pressure; dBP, diastolic blood pressure; bpm, beats per minute; DM, diabetes mellitus; PEM, pseudoexfoliation material; CI, confidence interval.

**Table 6:** Multivariate analysis for possible parameters associated with d peak amplitude.

Parameters	Estimate	95% CI	*p*-Value
Age, years	−3.0	−3.6, −2.4	<0.0001 **
Sex, F/M	−16	−31, −1.3	0.033 *
BMI, kg/m^2^	1.9	−0.024, 3.9	0.05
sBP, mmHg	0.66	0.16, 1.2	0.0091 **
dBP, mmHg	−2.0	−2.8, −1.2	<0.0001 **
Pulse rate, bpm	0.96	0.39, 1.5	0.0009 **
Hypertension, yes/no	−18	−34, −2.6	0.022 *
DM, yes/no	−6.4	−29, 16	0.58
PEM deposition, yes/no	−19	−39, −0.090	0.049 *
Glaucoma, yes/no	12	−9.0, 33	0.26

*p*-values were calculated using the generalized regression model. ** *p* < 0.01, * *p* < 0.05. BMI, body mass index; sBP, systolic blood pressure; dBP, diastolic blood pressure; bpm, beats per minute; DM, diabetes mellitus; PEM, pseudoexfoliation material; CI, confidence interval.

**Table 7:** Multivariate analysis for possible parameters associated with e peak amplitude.

Parameters	Estimate	95% CI	*p*-Value
Age, years	−0.588	−0.87, −0.30	<0.0001 **
Sex, F/M	−5.41	−12, 1.4	0.12
BMI, kg/m^2^	1.25	0.19, 2.3	0.021 *
sBP, mmHg	−0.392	−0.64, −0.14	0.0019 **
dBP, mmHg	0.379	0.0010, 0.76	0.049 *
Pulse rate, bpm	0.587	0.33, 0.84	<0.0001 **
Hypertension, yes/no	−11.9	−19, −4.3	0.0022 **
DM, yes/no	2.50	−7.1, 12	0.61
PEM deposition, yes/no	6.99	−1.5, 15	0.11
Glaucoma, yes/no	12.0	−0.041, 24	0.05

*p*-values were calculated using the generalized regression model. ** *p* < 0.01, * *p* < 0.05. BMI, body mass index; sBP, systolic blood pressure; dBP, diastolic blood pressure; bpm, beats per minute; DM, diabetes mellitus; PEM, pseudoexfoliation material; CI, confidence interval.

**Table 8:** Comparison of vascular types between Glaucoma (+) and Glaucoma (−) groups.

Parameters	Glaucoma (+) and PEM (−)	Glaucoma (+) and PEM (+)	Glaucoma (−)
Type A	185 (36)	50 (27)	39 (41)
Type B	273 (53)	104 (56)	44(47)
Type C	57 (11)	32 (17)	11(12)

*p* = 0.044, calculated using the chi-square test. Data are presented as N (%).

**Table 9:** Multivariate analysis for possible parameters associated with vascular types.

Parameters	Estimate	*p*-Value
Age, years	0.05	<0.0001 **
Sex, F/M	0.33	<0.0001 **
BMI, kg/m^2^	−0.04	0.10
sBP, mmHg	0.004	0.47
dBP, mmHg	0.02	0.021 *
Pulse rate, bpm	−0.03	<0.0001 **
Hypertension, yes/no	0.13	0.12
DM, yes/no	−0.05	0.63
PEM deposition, yes/no	0.09	0.35
Glaucoma, yes/no	−0.14	0.25

*p*-values were calculated using ordinal logistic regression. ** *p* < 0.01, * *p* < 0.05. BMI, body mass index; sBP, systolic blood pressure; dBP, diastolic blood pressure; bpm, beats per minute; DM, diabetes mellitus; PEM, pseudoexfoliation material; CI, confidence interval.

## Data Availability

The raw data supporting the conclusions of this article will be made available by the authors on request for ethical reasons.

## Abbreviations

The following abbreviations are used in this manuscript:

FMD	flow-mediated dilation
PWV	pulse wave velocity
CAVI	cardio–ankle vascular index
AI	augmentation index
APG	acceleration plethysmography
POAG	primary open-angle glaucoma
EXG	exfoliation glaucoma
BMI	body mass index
BP	blood pressure
BMI	body mass index
BP	blood pressure
PEM	pseudoexfoliation material
DM	diabetes mellitus
CI	confidence interval
